# 

**Published:** 1891-12

**Authors:** 


### CONTENTS—DECEMBER, 1891.—VOL. VII, No. 6.

ORIGINAL COMMUNICATIONS—

Polypi of Middle Ear and Auditory Canal as a Cause of Chronic Supurative Otitis. By R. C. Hodges, M. D., Galveston ........ 199

Enteritis Simulating Acute Intestinal Obstruction. By Daniel Parker, M. D., Calvert, Texas ........ 204

How to Prevent After-Pains. By T. J. Bennett, M. D., Austin ........ 213

Cancer: What is It? Causes and Treatment. By C. S. Reeves, M. D., Lone Grove, Texas ........ 215

CORRESPONDENCE—

Fœtal Skeleton Passed per Rectum. W. P. Powell, M. D., Willis, Texas ........ 217

Screw Worms in the Nose. Letter from T. C. Osborn, M. D., Cleburne ........ 220

Screw Worms in the Nose. Letter from T. J. Turpin, M. Dr. Laredo ........ 221

SOCIETY NOTES—

Smith County Medical Society ........ 221

Austin District Medical Society ........ 223

EDITORIAL—

A Feast of Reason ........ 223

Dietetic Treatment of Disease ........ 222

The Banquet A. D. M. S. and Election of Officers ........ 225

Domestic Animals vs. Man ........ 226

A National Board of Health; Bill in Congress ........ 227

Greeting—New Year ........ 228

MEDICAL NEWS AND MISCELLANY ........ 228

PUBLISHER'S NOTES ........ 232

